# Improving Breviscapine Oral Bioavailability by Preparing Nanosuspensions, Liposomes and Phospholipid Complexes

**DOI:** 10.3390/pharmaceutics13020132

**Published:** 2021-01-20

**Authors:** Zilin Song, Jiaojiao Yin, Peifu Xiao, Jin Chen, Jingxin Gou, Yanjiao Wang, Yu Zhang, Tian Yin, Xing Tang, Haibing He

**Affiliations:** 1 Department of Pharmaceutics, College of Pharmacy, Shenyang Pharmaceutical University, Shenyang 110016, China; songzilin93@163.com (Z.S.); Yinjiaojiao0406@163.com (J.Y.); xiaopeifu96@163.com (P.X.); chenjin951102@126.com (J.C.); paladin.13@163.com (J.G.); wang53k2002@163.com (Y.W.); zhangyu@syphu.edu.cn (Y.Z.); tanglab@126.com (X.T.); 2 School of Food and Wine, Shenyang Pharmaceutical University, 103 Wenhua Road, Shenyang 110016, China; yintian124@vip.163.com

**Keywords:** breviscapine, oral bioavailability, nanosuspensions, liposomes, phospholipid complexes

## Abstract

Breviscapine (BVP), a flavonoid compound, is widely used in the treatment of cardiovascular and cerebrovascular diseases; however, the low oral bioavailability and short half-life properties limit its application. The aim of this study was to investigate the three preparations for improving its oral bioavailability: nanosuspensions (BVP-NS), liposomes (BVP-LP) and phospholipid complexes (BVP-PLC). In vitro and in vivo results suggested that these three could all significantly improved the cumulative released amount and oral bioavailability compared with physical mixture, in which BVP-PLC was the most optimal preparation with the relative bioavailability and mean retention time of 10.79 ± 0.25 (*p* < 0.01) and 471.32% (*p* < 0.01), respectively. Furthermore, the influence of drug-lipid ratios on the in vitro release and pharmacokinetic behavior of BVP-PLC was also studied and the results showed that 1:2 drug-lipid ratio was the most satisfactory one attributed to the moderate-intensity interaction between drug and phospholipid which could balance the drug loading and drug release very well.

## 1. Introduction

**Figure 1:**
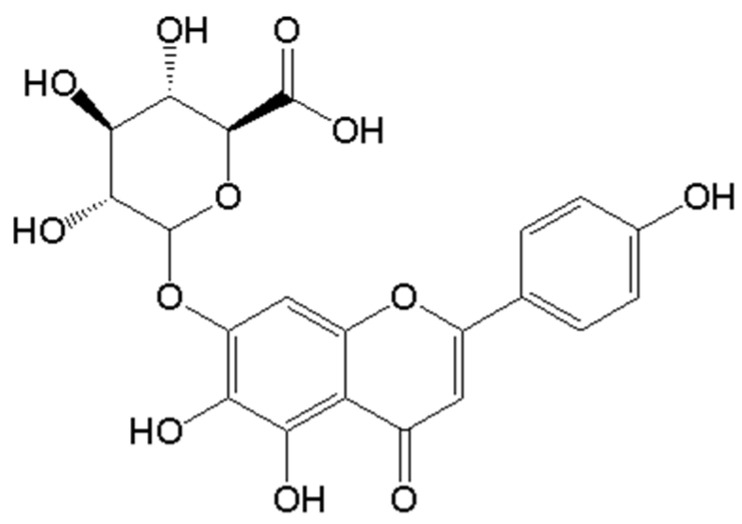
The structure of scutellarin.

Breviscapine (BVP) is a flavonoid compound extracted from the Chinese herb Erigeron breviscapus (Vant) Hand-Mazz. Scutellarin (>85.0%), 4,5,6-tetrahydroxyflavone-7-*O*-glucuronide (Figure 1), is a flavonoid glycoside compound and is the main active ingredient of BVP [1]. BVP has various pharmacological activities, including dilating blood vessels, increasing cardiovascular and cerebrovascular blood flow, anti-platelet aggregation and improving microcirculation [2,3], anti-inflammatory [4], renoprotective and neuroprotective effects [1,5,6,7,8]. Therefore, BVP products are widely used in the treatment of cardiovascular and cerebrovascular diseases such as coronary artery disease, myocardial infarction, hypertension, hyperlipidemia and diabetic complications in China [9]. Cerebrovascular and cardiovascular diseases are chronic diseases which require drugs with good bioavailability and long half-life. Unfortunately, BVP belongs to the Biopharmaceutical Classification System (BCS) class Ⅳ drugs and its half-life is very short [10,11]. Studies have shown that there are several main causes of the low bioavailability of BVP: (1) low oral solubility, limited membrane permeability, transporter mediated drug efflux [12] and first-pass elimination in gastrointestinal tract (GIT) [13,14]; (2) the hydroxyl groups in 5, 6, 7 and 4′ positions of BVP can combine with endogenous-α-d-glucuronic acid to form glucuronic glycosides which results rapidly metabolized [15]; (3) metabolic enzymes and pH-mediated environmental degradation in GIT; (4) wide distribution in body and diversity of biological transformation [16,17]. In recent years, researchers have prepared various formulations to improve the oral bioavailability of BVP by using nanoemulsions, nanoparticles, liposome nanocomposite particles, phospholipid complexes and more [18,19,20].

Nanotechnology is an effective tool for improving the bioavailability and bioactivity of many water-insoluble drugs, for example flavonoids, as it can endow the preparations with reduced particle sizes [21]. Nanosuspensions are defined as a carrier-free drug delivery system usually consisting of a stabilizer and active pharmaceutical ingredient (API), which possesses high drug loading capacity and satisfactory biocompatibility [22]. Liposomes are a kind of spherical vesicles which have biofilm-like bilayer consisting of phospholipids and cholesterols and so forth. They have a size range from ~20 nm to ~5 µm in diameter, with one or more lipid bilayers surrounding aqueous compartments [23]. Liposomes have several desirable properties, such as good biodegradability and biocompatibility, prolonged release effect and enhanced chemical stability of drugs [24,25,26,27,28,29,30,31]. Phospholipid complexes, also named phytosomes, are a novel drug delivery system formed by non-covalent bonds (electrostatic interaction, van der Waals force, hydrogen bond and etc.) between phospholipids and drugs [32].The structure of phospholipid complexes is similar to that of liposomes, with the major difference being that in phospholipid complexes, the drug is mainly connected with the phospholipid polar head, whereas in liposomes, the drug is encapsulated in its cavity or phospholipid bilayer [33]. Drug-phospholipid complexes possess amphiphilic characteristics, which makes them more readily soluble in GIT and more easily absorbed through amphiphilic cell membrane and lymphatic system, thereby improving the bioavailability of drugs. Furthermore, phospholipid complexes can increase drug stability, extend the action duration of drugs and delay drug elimination [32]. Last several years, phospholipid complexes have been widely used to improve oral bioavailability of poorly water-soluble drugs, for example quercetin [34], kaempferol [35], silybin [36], curcumin [37] and tamoxifen [38].

**Figure 2:**
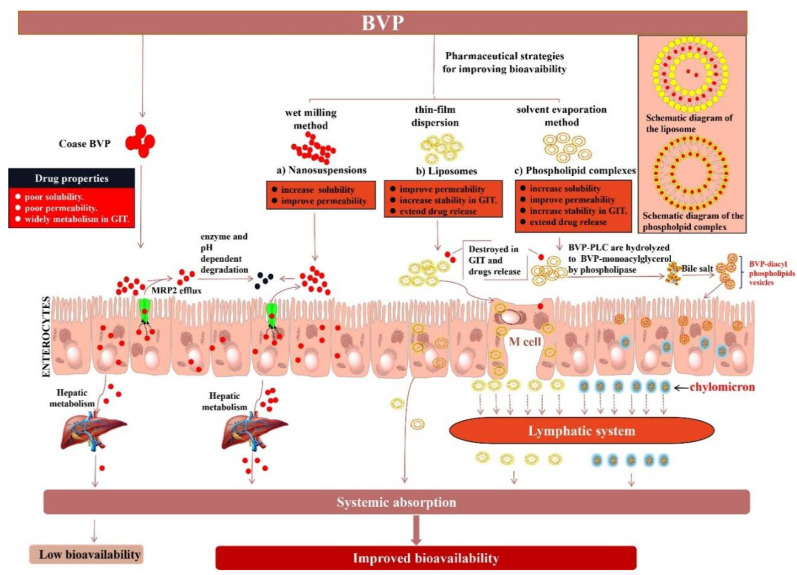
The outline of this paper and the mechanism of Breviscapine–nanosuspension (BVP–NS), BVP–liposomes (LP) and BVP–phospholipid complexes (PLC) to enhance bioavailability.

In this study, three preparations, breviscapine nanosuspensions (BVP-NS), breviscapine liposomes (BVP-LP) and breviscapine phospholipid complexes (BVP-PLC) were prepared for oral administration to increase the drug absorption and extend the half-life in order to improve bioavailability. The particle size and zeta potential were measured by dynamic light scattering (DLS), besides the morphology was observed by use of transmission electron microscopy (TEM), the results indicated that the particle sizes were about 200–300 nm and zeta potentials were all negative. The existence form of BVP in different preparations was investigated by polarized light microscopy (PLM), differential scanning calorimetry (DSC) and powder X-ray diffraction (PXRD), which suggested that BVP was existed in crystal form in NS, while as non-crystal form in LP and PLC. An FT-IR experiment was carried out to investigated the interactions between BVP and phospholipid molecule, the results demonstrated there really existed interactions. Molecule simulation further indicated the –OH and –COO^−^ group in BVP could form hydrogen bond and electrostatic interaction with phospholipid molecule in PLC, rather than NS and LP. In vitro release results manifested that BVP-PLC showed the highest cumulative release amount of, followed by BVP-NS and BVP-LP. In vivo pharmacokinetic study verified that BVP-NS, BVP-LP and BVP-PLC all shown significantly higher oral bioavailability compared with BVP. In addition, BVP-LP and BVP-PLC exhibited a distinctly extended-release profile. Furthermore, the drug-lipid ratio influence on the pharmacokinetic parameters of BVP-PLC was also investigated, in which the preparation with drug-lipid ratio of 1:2 had the highest relative bioavailability. As the higher drug-lipid ratio (1:1) could not provide strong enough force to maintain the stability of the preparation and the lower drug-lipid ratio (1:3) impacted the dissociation of BVP from the preparation. Therefore, phospholipid complexes with appropriate drug-lipid ratio provide an effective strategy for improving the oral bioavailability of drugs which have low solubility both in water and lipid. The Figure 2 illustrated overall scheme of this work and the mechanism of the three preparations to improve bioavailability.

## 2. Materials and Methods

### 2.1. Materials and Animals

BVP (scutellarin purity > 98.5%) was purchased from Kunming Longjin Pharmaceutical Co., Ltd. (Yunnan, China). Soybean phospholipid was provided by Shenyang Tianfeng Bio-Pharmaceutical Co., Ltd. (Shenyang, China). Hydroxypropyl methylcellulose E5 (HPMC-E5) was obtained from Dow Chemicals Company (Midland, MI, USA). Scutellarin reference substance and baicalin (internal standard, IS) were provided by National Institute of Food and Drug Control (Beijing, China). Mannitol was supplied by Tianjin Heng Xing Chemical Reagent Co., Ltd. (Tianjin, China). Cholesterol was supplied by Hennan Liwei Biological Pharmaceutical Co., Ltd. (Jiaozuo, China). Vitamin E was provided by Zhejiang NHU Company Co., Ltd. (Xinchang, China). All other chemicals and reagents used were of analytical or chromatographic quality and deionized water was used throughout the study.

Male Sprague-Dawley rats were provided by Liaoning Changsheng Biotechnology Co., Ltd. (Shenyang, China). All the animal research was approved by the Committee of Ethics of Animal Experimentation of Shenyang Pharmaceutical University and carried out in accordance with the guidelines of this committee (SYPU-IACUC-C2019-7-3-204).

### 2.2. Preparation of BVP-NS, BVP-LP and BVP-PLC

#### 2.2.1. BVP-NS

The BVP-NS were prepared by wet-milling method [39]. BVP (3.0 g) and HPMC-E5 (0.6 g) were weighed and dispersed in a small amount of distilled water. Separately, soybean lecithin (6.0 g) was dissolved in 7.5 mL of absolute ethanol and stirring (DF-101S, Gong yi yu hua Instrument Co. LTD, Gongyi, China) at 60 °C, after the solution turn into yellow and clear, the water phase was added in and then the mixture was diluted by distilled water to 150 mL. Then, transferred into a Superfine Grinding Equipment (Mini-easy, Retsch Topway Technology Co., Ltd., Beijing, China) with zirconium oxide milling beads (0.6–0.8 mm diameter) as the milling media and milling for 2 h at 2000 rpm. The final suspension was spray-dried to obtain a solid dosage form with 2.5% mannitol as protective agent.

#### 2.2.2. BVP-LP

The thin-film dispersion method was used to prepare BVP-LP [24,25,27,28]. 1.0 g phospholipid and 0.25 g cholesterol were accurately weighed and dissolved in absolute ethanol. The organic solvent was evaporated at 50 °C for 30 min to form a thin film and then hydrated using 100.0 mL of phosphate buffer solution (pH 7.4) containing 200.0 mg BVP for 1 h at 55 °C until all the lipids were completely dispersed into the aqueous phase. The crude liposome dispersion was subjected to ultrasound for 3 min (on 2.5 s, off 3 s) by a SCIENTZ-IID Ultrasonic Cell Crusher (Xinzhi Biological Technology Co., Ltd., Ningbo, China) at 280 W in ice bath. After the free BVP was removed as method described in 2.4, the final liposome dispersion obtained. Next, 10% (*w*/*v*) mannitol as the lyoprotectants were added and filtered through a 0.22 µm filter to remove insoluble impurities. The filtered preparations were lyophilized at −20 °C for 24 h to obtain the freeze-dried BVP-LP powder. The blank liposome was prepared by the same method except that BVP was not added.

#### 2.2.3. BVP-PLC

BVP-PLC was prepared by a solvent evaporation method [32]. Briefly, BVP (0.60 g) and appropriate phospholipid were weighed at different mass ratios (1:1, 1:2, 1:3) and dissolved in 150 mL absolute ethanol in a 250 mL round bottom flask, with appropriate amount of Vitamin E added in as antioxidant. The mixture was refluxed at 50 °C for 2 h. Subsequently, the resultant clear solution was completely evaporated by spray drying with 2.5% mannitol as protective agent.

### 2.3. Encapsulation Efficiency (EE) of BVP-LP

The liposomes and free BVP were isolated by ultrafiltration. 4.0 mL preparation was accurately measured and placed in an ultrafiltration tube with cutoff molecular weight of 100 kDa and centrifuged at 3000 rpm for 20 min. The encapsulated BVP-LP remained on the surface of the filter membrane and free drug was collected into the bottom of the filter [40]. The separated bottom phases were transferred to 10 mL volumetric flask and diluted to the volume with methanol. After filtration by a 0.22 μm microporous filtration membrane, the samples and reference solution were qualified by HPLC (Agilent 1260 L, C18 column (Hypersil GOLD C18, 250 × 4.6 mm^2^, 5 μm, Thermo, USA), UV detector (set at 335 nm, Agilent Technologies Inc., California, USA) and the mobile phase was methanol/0.1% phosphoric acid solution (40:60, *v/v*) with a flow rate of 1.0 mL/min. The EE of BVP-LP was determined by Equation (1).

(1) $$EE=\frac{The\;mass\;of\;total\;BVP-the\;mass\;of\;free\;BVP}{The\;mass\;of\;total\;BVP}\times 100\%$$

### 2.4. Complexation Efficiency (CE) of BVP-PLC

The CE of phospholipid complexes is an important parameter. Due to the different solubilities in chloroform, the CE of phospholipid complexes could be measured, in which free BVP is insoluble but phospholipid complexes are readily soluble [36]. The phospholipid complexes (equivalent to 10.0 mg of BVP) was accurately weighed and then dissolved in 10.0 mL chloroform and sonicated for 30 min. After centrifugation at 3000 rpm for 5 min, the supernatant was collected and filtered using a 0.22 μm filter. 1.0 mL of the filtrate was accurately measured and transferred into a 50 mL round bottom flask. After the chloroform removed by rotary evaporation, appropriate amount of methanol was added to dissolve the precipitation. Next, the mixture was transferred into a 10 mL volumetric flask and diluted to the volume with methanol. The amount of BVP encapsulated was determined by HPLC as above mentioned. In order to obtain the total amount of BVP, the phospholipid complexes (equivalent to 10.0 mg of BVP) was dissolved by appropriate amount of methanol and transferred into a 10 mL volumetric flask and diluted to the volume with methanol and determined as above described. The complexation efficiency (CE) was defined by Equation (2).

(2) $$CE=\frac{The\;amount\;f\;combined\;BVP}{The\;amount\;of\;total\;BVP}\times 100\%$$

### 2.5. Characterization

#### 2.5.1. Determination of Particle Size and Zeta Potential

Mean particle size, polydispersity index (PDI) and zeta potential of BVP-NS, BVP- LP and BVP-PLC were measured using Zeta sizer nano-ZS 900 (Malvern Instruments, Malvern, UK) (medium:water; dispersant refractive index(RI): 1.33cP; Viscosity: 0.8872; dielectric constant: 78.36 F/m; count rate: 200–400; material RI: 1.59; wavelength: 633 nm). BVP-NS, BVP-LP or BVP-PLC were dispersed in purified water and diluted to an appropriate concentration. Each sample was measured in triplicate at room temperature and the result was expressed as mean ± SD.

#### 2.5.2. Polarized Light Microscopy (PLM) Analysis

PLM was used to characterize the BVP-NS, BVP-LP and BVP-PLC (1:2). The mannitol, HPMC-E5, soybean lecithin, cholesterol, BVP, BVP-NS, BVP-LP, BVP-PLC (1:2) and three physical mixtures (the first (PM-BVP-NS) consisting of BVP, soybean lecithin, HPMC-E5 and mannitol; the second (PM-BVP-LP) consisting of BVP and blank liposome; the third (PM-BVP-PLC (1:2)) consisting of BVP, phospholipid and mannitol) were observed using positive polarizing microscope (Leica DM2700P, Leica Microsystems, Wetzlar, Germany) at a 90° angle between the lifting mirror and the detecting mirror (vertical).

#### 2.5.3. Transmission Electron Microscopy (TEM) Analysis

BVP-NS, BVP-LP and BVP-PLC (1:2) were negatively stained with 2% phosphotungstic acid and placed on a copper grid coated with carbon film and then the morphologies were observed using TEM (Hitachi HT7700, Hitachi, Ltd., Tokyo, Japan).

#### 2.5.4. Fourier Transform Infrared Spectroscopy (FT-IR) Analysis

The samples of mannitol, HPMC-E5, soybean lecithin, cholesterol, BVP, PM-BVP-NS, BVP-NS, PM-BVP-LP, BVP-LP, PM-BVP-PLC (1:2) and BVP-PLC (1:2) were prepared by KBr pressing method for the measurement by Fourier transform infrared (FTIR) spectrophotometer (EQUINOX 55, Bruker, Karlsruhe, Germany).

#### 2.5.5. Differential Scanning Calorimetry (DSC) Analysis

The thermal behavior of mannitol, HPMC-E5, soybean lecithin, cholesterol, BVP, PM-BVP-NS, BVP-NS, PM-BVP-LP, BVP-LP, PM-BVP-PLC (1:2) and BVP-PLC (1:2) were analyzed using DSC-3 (METTLER TOLEDO, Zurich, Switzerland). The samples were sealed in crimped standard aluminum pans and heated under nitrogen flow (50 mL/min) at a rate of 10 °C/min from 30 °C to 300 °C.

#### 2.5.6. Powder X-ray Diffraction (PXRD) Analysis

The polymorphic state of mannitol, HPMC-E5, soybean lecithin, cholesterol, BVP, PM-BVP-NS, BVP-NS, PM-BVP-LP, BVP-LP, PM-BVP-PLC (1:2) and BVP-PLC (1:2) were determined by a D/Max-2400 X-ray diffractometer (Rigaku, Osaka, Japan). A continuous scan mode was run with a rate of 2°/min over a 2θ range from 5° to 60° and data were collected with Cu-Ka radiation and a voltage of 56 kV.

### 2.6. In Vitro Release Studies

The in vitro release of BVP-NS, BVP-LP and BVP-PLC were carried out by ZRS-8G dissolution apparatus (Tianjin Tianda Tianfa Technology Co., Ltd, Tianjin, China). The dissolution medium was pH 6.8 phosphate buffer solution, the rotation speed was 50 rpm and the temperature was 37 ± 0.5 °C. Predetermined amounts of BVP-NS, BVP-LP or BVP-PLC (drug mass approximately 36 mg) were added in 900 mL dissolution medium. 5 mL release medium was sampled at the time point of 5, 15, 30, 45, 60, 90 and 120 min and 5 mL of fresh dissolution medium was added to maintain sink condition. The content was determined by HPLC as previous described and the cumulative dissolution percentage of the drug at each time point was calculated using the external standard method.

### 2.7. Pharmacokinetic Studies

In order to compare the oral bioavailability of BVP in the three formulation strategies, the pharmacokinetic study was performed. Male Sprague–Dawley rats weighing from 200 to 220 g were randomly divided into four groups (*n* = 4). Each drug solution in the different formulations (the formulations were dispersed with 0.5% sodium carboxymethyl cellulose) was administered to rats by gavage (20 mg/kg BVP): Group A (BVP), Group B (BVP-PLC), Group C (BVP-NS) and Group D (BVP-LP (1:2)). Prior to the experiment, the SD rats were fasted for 12 h but had free access to water. After gavage administration, 0.5 mL blood were sampled at 0.25, 0.5, 1, 2, 4, 6, 8, 10, 12, 24 h and transferred into a heparinized centrifuge tube. The collected samples were centrifuged at 5000 rpm for 10 min at 4 °C and the supernatant plasma was analyzed by UPLC-MS/MS (Waters Corp, Manchester, UK). Food was provided to the rats 4 h after of administration. At the same time, a pharmacokinetic study was also performed to compare the oral bioavailability of BVP-PLC with different drug-lipid ratios (1:1, 1:2, 1:3).

The plasma concentration of BVP was measured using ultrahigh performance liquid chromatography tandem mass spectrometry (UPLC–MS/MS) [41]. An ACQUITY UPLC system (Waters Corp., Milford, MA, USA) and Thermo C18 column (50 mm × 2.1 mm ID, 1.7 μm, Thermo Fisher SCIENTIFIC, Thermo, USA) was used for the chromatographic separations, with a gradient elution of methanol and 0.1% formic acid solution (Table 1). Mass spectrometric determination was performed in positive ESI mode and the compounds were quantified by multiple reaction monitoring (MRM, Waters Corp, Manchester, UK) of the transitions of m/z 463→287.1 for BVP and m/z 447.1→271.1 for Baicalin as IS, respectively.

### 2.8. Statistical Analysis

All data are expressed as the mean and standard deviation (mean ± SD). The statistical significance of the differences between the groups was assessed by non-parametric statistics—Kruskal-Wallis ANOVA with post-hoc test. Analysis of variance was used for comparing more than two samples, where *p* < 0.05 and *p* < 0.01 (two-tailed) were considered statistically significant and extremely significant, respectively. Pharmacokinetic parameters were obtained with a non-compartmental method using drug and statistics (DAS) software.

## 3. Results and Discussion

### 3.1. Characterization

#### 3.1.1. Particle Size and Zeta Potential

The reduction of drug particle size into nanoscale is an effective method to enhance oral bioavailability. However, with the decrease of particle size, the increase of surface energy would lead to particle aggregation and deposition. The addition of stabilizers and an appropriate zeta potential are beneficial to the stability of the preparation. The particle size, PDI and zeta potential of three preparations were summarized in Table 2. The particle size of all three preparations was about 300 nm [42,43] and the PDI of three preparations were all smaller than 0.3. The zeta potentials were all negative, which could provide effective prevention of aggregation of particles to maintain a homogenous preparation. It was worthy to note that the particle size of BVP-PLC tended to increase with the decrease of the drug-lipid ratio, which may be caused by the excessive phospholipids attached to the phospholipid complexes.

#### 3.1.2. EE (%) of BVP-LP and CE (%) of BVP-PLC

Since BVP is a weak acidic drug, unencapsulated BVP could dissolved in weak alkaline solution and therefore the EE was determined by ultrafiltration centrifugation method. The EE of BVP-LP with SPC/Chol weight ratio of 4:1 was 76.41 ± 5.61% (Table 2). The CE represents an extremely important index for prescription screening of BVP-PLC. The determination of the CE is based on different solubility of formed phospholipid complexes and the free drug in chloroform, in which the phospholipid complexes could readily dissolve while the free BVP remains highly insoluble [44]. As shown in Table 2, the CE of BVP-PLC increased as the drug-lipid ratio decreasing, with the highest of 79.36 ± 3.76% at drug-lipid ratio of 1:3.

#### 3.1.3. TEM

**Figure 3:**
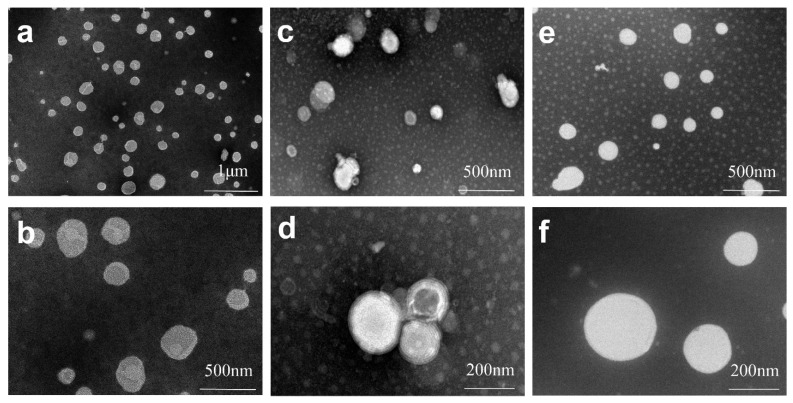
Transmission electron microscopy (TEM) images of BVP-NS (**a**,**b**), BVP-LP(**c**,**d**) and BVP-PLC (1:2) (**e**,**f**).

The morphologies of BVP-NS, BVP-LP and BVP-PLC (1:2) observed by transmission electron microscopy (TEM) were shown in Figure 3. The results indicated that all samples exhibited a spherical shape. It is well known that when phospholipids are dispersed in an aqueous solution, they can self-assemble to form ordered structures such as micelles, vesicles, liposomes, bilayer membranes and planar phospholipid multilayer membranes according to the different environments (for example, different concentrations of phospholipids). For BVP-NS, a large amount of soybean phospholipid was added as stabilizer. The majority of drugs was encapsulated by phospholipids and could form spherical particles after wet grinding (Figure 3a,b). For BVP-LP, the morphology a little differed from BVP-NS and BVP-PLC (1:2), which presented a fingerprint-like internal structure with a size around 200 nm (Figure 3c,d). The TEM images revealed the morphology of the BVP-PLC (1:2) was in globular shape with no internal structures, suggesting that the actual structure of BVP-PLC may be vesicles or micelles (Figure 3e,f). Anisha Mazumder et al. also confirmed that sinigrin-phytosome complexes formed vesicle-like structures which were ellipsoidal or spherical, self-closed, well-identified, well-formed and dispersed by TEM [45].

#### 3.1.4. PLM Analysis

**Figure 4:**
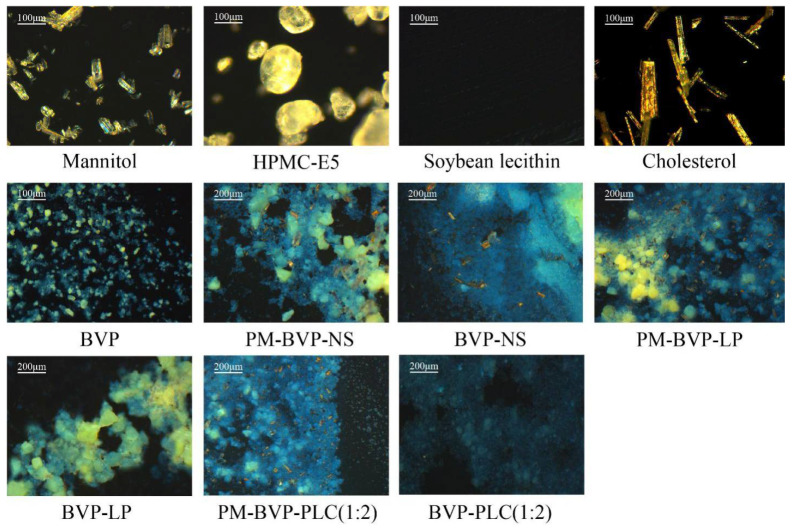
Polarized light microscopy (PLM) images of mannitol, HPMC-E5, soybean lecithin, cholesterol, BVP, PM-BVP-NS, BVP-NS, PM-BVP-LP, BVP-LP, PM-BVP-PLC (1:2) and BVP-PLC (1:2).

Polarized light microscopy (PLM) was often used to evaluate the birefringence phenomenon of crystal drugs, amorphous drugs and polymer materials [43,46]. If the matters were amorphous, there will be no birefringence and the field of vision will be dark. PLM results of ingredients and different formulations were shown in Figure 4. The ingredients of mannitol, HPMC-E5, cholesterol and BVP showed significant birefringence phenomenon except for soybean lecithin which is amorphous and therefor the field is dark. For BVP-NS, the birefringence phenomenon of BVP still existed but a little weak compared with PM-BVP-NS, indicated that most BVP was still in crystal form but part of the drug was transformed into an amorphous form during nano-milling and/or the spray drying process [46]. The result of PM-BVP-LP (1:2) showed distinct birefringence phenomenon of mannitol, cholesterol and BVP, while in BVP-LP, the BVP signal weakened or even disappeared, indicating that the drug was not exist in the form of crystal in liposomes. According to the property of BVP and structure of liposome, the drug was very likely to exist in the form of molecules between the lipid bilayer or in the internal aqueous phase. The result of BVP-PLC (1:2) almost had no BVP signal compared with PM-BVP-PLC (1:2), indicating that the drug in BVP-PLC (1:2) was also not in crystal form, which may present in a highly dispersed molecular state.

#### 3.1.5. FT-IR Analysis

**Figure 5:**
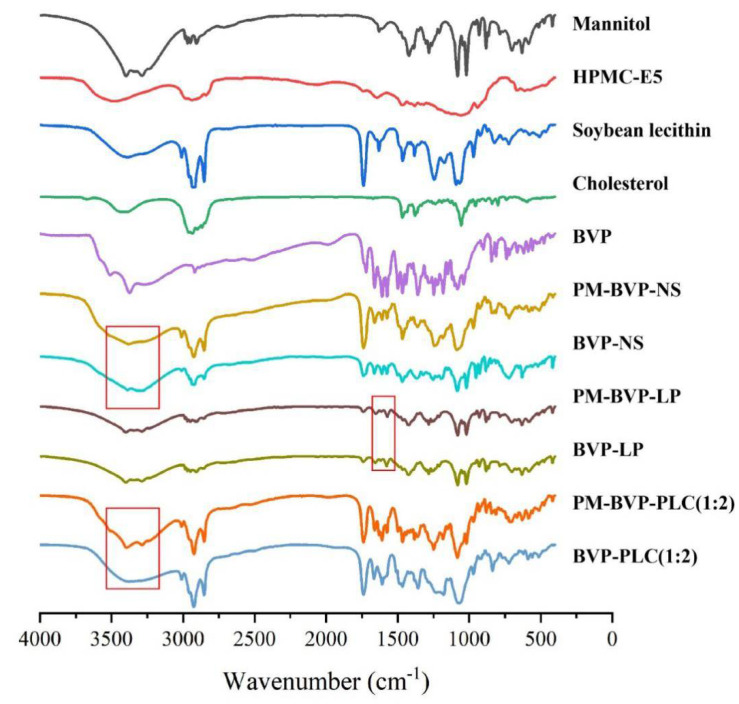
Fourier transform infrared (FTIR) spectrum of mannitol, HPMC-E5, soybean lecithin, cholesterol, BVP, PM-BVP-NS, BVP-NS, PM-BVP-LP, BVP-LP, PM-BVP-PLC (1:2) and BVP-PLC (1:2).

Fourier transform infrared spectroscopy (FT-IR) is an effective method to characterize molecular structure and is widely used to study molecular interactions between drugs and excipients. As shown in Figure 5, the BVP exhibited characteristic FT-IR absorption peaks at 3509.6, 3373.8 and 3275.5 cm^−1^ (–OH stretching vibration), 1720.9 cm^−1^ (C=O stretching vibration), 1661.3 cm^−1^ and 1600 cm^−1^ (C=C stretching vibration), 1360.4 cm^−1^ and 1083.0 cm^−1^ (C–O deformation vibration) and 1222.8 cm^−1^ (C–O–H stretching vibration). For BVP-NS and BVP-LP, all characteristic BVP peaks were presented in spectra compared with PM-BVP-NS and PM-BVP-LP, indicating that BVP in the BVP-NS or BVP-LP maintains the original drug structure and there was no detectable interaction.

As for BVP-PLC (1:2), the FT-IR spectra showed a bimodal infrared absorption at 3395.7 and 3286.1 cm^−1^, which were absorption peaks of hydroxyl and carboxyl groups in the range of 3650–3200 cm^−1^ and was superimposable with pure BVP and excipients. However, compared with BVP and PM-BVP-PLC (1:2), the BVP-PLC (1:2) had one strong wider absorption peak in the range of 3650–3200 cm^−1^ and meanwhile shifting to lower wave number, suggesting that the hydroxyl group in BVP could form interactions with phospholipid molecules during complexes formation [33]. These findings were in agreement with diosmin lyophilized nano-phytosome [47] described in the literature, where weak interaction between diosmin and phospholipid formed during the preparation of the complexes.

**Figure 6:**
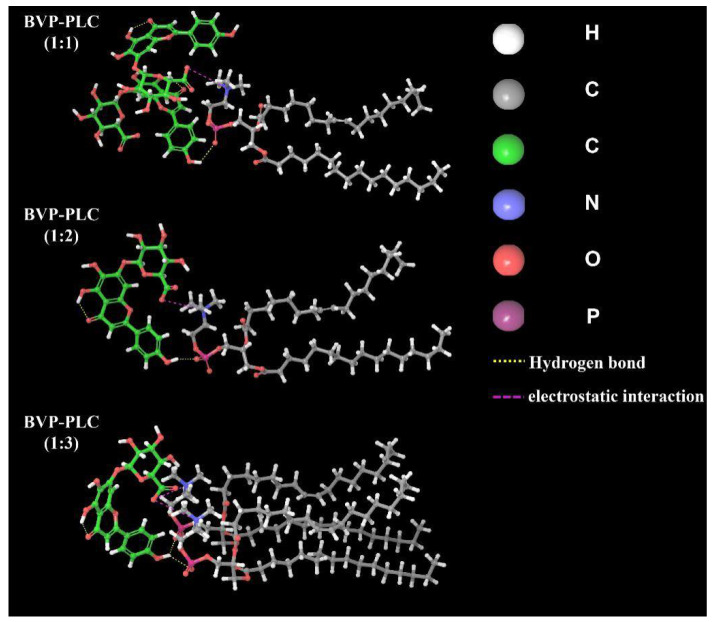
Interaction between drugs and phospholipids in BVP-PLC with different drug-lipid ratios.

Furthermore, from the perspective of molecular simulation, we could apparently observe the interaction between carboxyl and hydroxyl groups in BVP and phospholipid molecules. As the most representative substance in soybean lecithin, phosphatidyl cholines (PC, >60%) was used as molecular simulation model to study the interactions between drug and phospholipid in BVP-PLC with different drug-lipid ratios. As shown in Figure 6, the interaction forces between drug and phospholipid were mainly two types, hydrogen bond (–OH group in drug and –P=O group in phospholipid) and electrostatic interaction (–COO^−^ group in drug and –N^+^ in phospholipid) [32,33]. In addition to strength, the interaction forms also varied with the drug-lipid ratio. For BVP-PLC (1:1), there were about two molecules of BVP interact with phospholipid, the compact forces were relatively weak. When the drug-lipid ratio raised to 1:2, the molar ratio of phospholipid to drug was about 1:1, one phospholipid molecule compressed one molecule BVP, interaction was strengthened. As for drug-lipid ratio of 1:3 or even lower, the phospholipid in preparation was so overwhelmed that the encapsulation forces were further enhanced, for about two or even more phospholipid molecules to compress one BVP molecule.

#### 3.1.6. DSC Analysis

**Figure 7:**
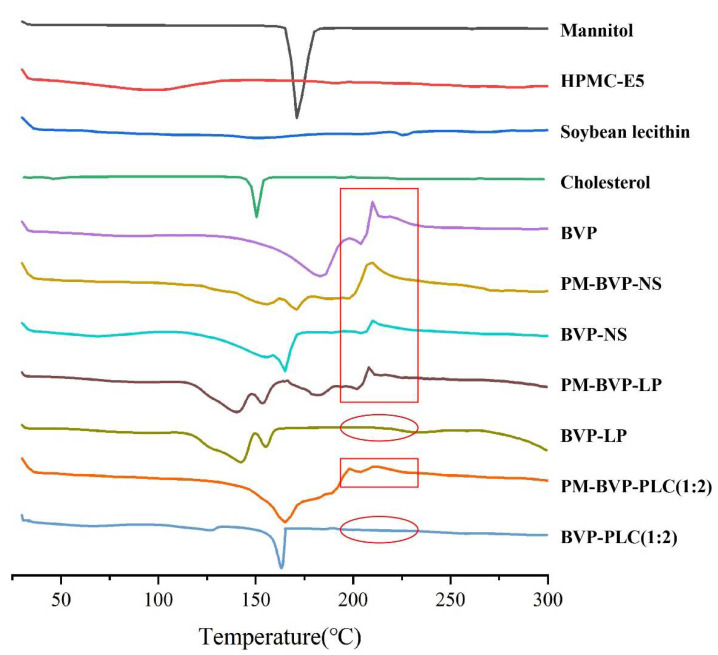
Differential scanning calorimetry (DSC) thermograms of mannitol, HPMC-E5, soybean lecithin, cholesterol, BVP, PM-BVP-NS, BVP-NS, PM-BVP-LP, BVP-LP, PM-BVP-PLC (1:2) and BVP-PLC (1:2).

The differential scanning calorimetry (DSC) was also used to determine the exist form of BVP in different preparations. As shown in Figure 7, the thermograms of mannitol and cholesterol exhibited a sharp endothermic peak at 166.1 °C and 150.3 °C, respectively. BVP showed an endothermic peak at 182.1 °C and an exothermic peak at 210.3 °C, while HPMC-E5 and soybean lecithin had no obvious endothermic and exothermic peak.

The DSC curve of BVP-NS was slightly changed compared with BVP and PM-BVP-NS. The characteristic exothermic melting peaks of BVP were still present and the endothermic peaks of BVP was superimposed with mannitol to form a blunt peak in PM-BVP-NS and BVP-NS. The biggest difference for BVP-NS was that the enthalpy at 210.3 °C was decreased compared with PM-BVP-NS, which indicated that some BVP was transformed into an amorphous state during the milling-drying process [22] and this result can also correspond to PLM. Compared with BVP and PM-BVP-LP, the characteristic peaks of BVP disappeared in the DSC curve of BVP-LP indicated that the BVP existed in non-crystal form. Of note, the peaks of cholesterol and mannitol shifted to 142.3 °C and 155.2 °C because of lyophilization, respectively.

While for the BVP-PLC (1:2), the DSC curve of BVP-PLC (1:2) was greatly changed compared with BVP and PM-BVP-PLC (1:2). In the PM-BVP-PLC (1:2) spectrum, the peaks were the simple overlap of BVP, mannitol and soybean lecithin. Whereas in BVP-PLC (1:2), the characteristic absorption melting peaks of BVP at 182 °C and 210 °C disappeared and instead showed a sharp absorption peak at 165.34 °C (mannitol characteristic peak), indicating that BVP-PLC (1:2) formed by BVP and phospholipids was not a simple physical mixture but a new binding state.

#### 3.1.7. PXRD Analysis

**Figure 8:**
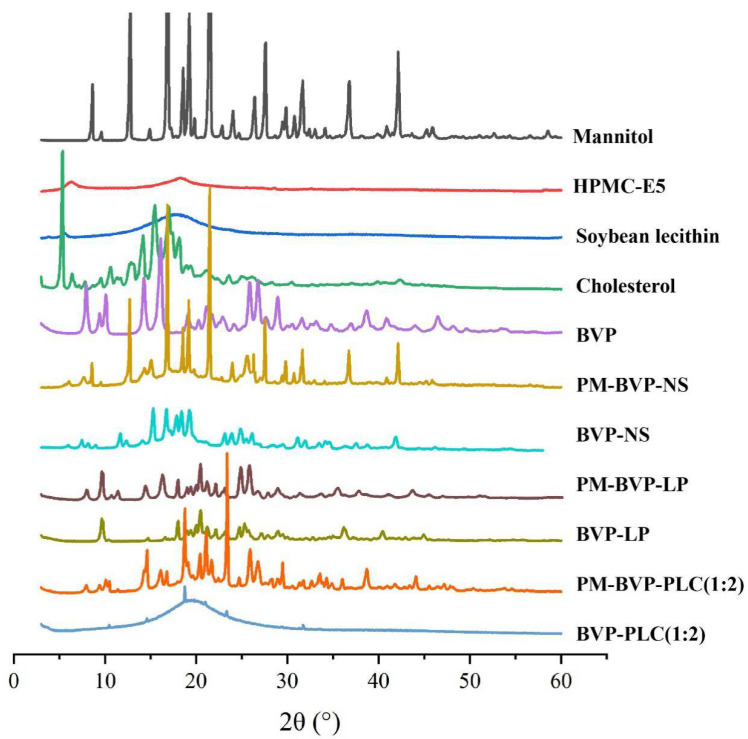
Powder X-ray diffraction (PXRD) pattern of mannitol, HPMC-E5, soybean lecithin, cholesterol, BVP, PM-BVP-NS, BVP-NS, PM-BVP-LP, BVP-LP, PM-BVP-PLC (1:2) and BVP-PLC (1:2).

To further confirm the crystalline state of BVP in BVP-NS, BVP-LP and BVP-PLC (1:2), PXRD analysis was conducted and the X-ray diffractograms were shown in Figure 8. For BVP, characteristic sharp peaks were seen at 2θ of 7.94°, 9.40°, 10.10°, 14.27°, 16.11°, 21.25.82°, 26.78°, 28.94° and 38.70°. As for the PM-BVP-NS and BVP-NS, the typical crystal peaks of the of BVP were still appeared but with a little decreased in intensity. This further indicated that most of BVP in NS preparation remained in a crystalline form, while some of transferred to amorphous after wet grinding and spray drying, which was agreement with the PLM and DSC results. For the BVP-LP and BVP-PLC (1:2), the characteristic crystal peaks of BVP disappeared, indicating that BVP in BVP-LP and BVP-PLC (1:2) was highly dispersed in the form of molecules or amorphous morphology, which could validate with pervious conclusion.

### 3.2. In Vitro Release Studies

**Figure 9:**
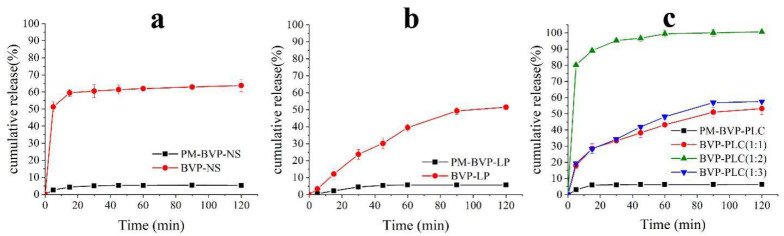
The cumulative release curves of BVP-NS (**a**), BVP-LP (**b**) and BVP-PLC (**c**).

In vitro release study is a very important evidence to predict the in vivo absorption profile. As shown in Figure 9a, the total dissolution amount of the PM-BVP-NS was less than 7% until 120 min, while BVP-NS exhibited higher and quicker cumulative release profile with the final release amount of 63.77 ± 3.58%, which was mainly attributed to the decrease in particle size to the nanometer range and the increased surface area [22].

The in vitro release profiles of BVP-LP and PM-BVP-LP were depicted in Figure 9b. The release of the physical mixture was very slow and the final amount in 2 h was less than 7%. After preparing BVP into liposomes, the in vitro release was improved and extended with the final release amount reached 51.50 ± 1.32%, which confirmed that preparing BVP into liposomes could improve the drug release, to a certain extent.

Figure 9c showed the in vitro release profiles of PM-BVP-PLC and BVP-PLC. The dissolution behavior of the physical mixture was similar with that of PM-BVP-NS for about 7%. Notably, compared to the PM-BVP-PLC, the BVP-PLC showed a dramatic enhancement of the rate and extent of dissolution, no matter which drug-lipid ratio. In specific, the dissolution behaviors of the BVP-PLC with drug-lipid ratios of 1:1 and 1:3 were similar, while the latter was slightly better than that of the former, with a cumulative release of 53.22 ± 3.58% and 58.53 ± 0.85%, respectively. Obviously, the BVP-PLC with a drug-lipid ratio of 1:2 exhibited the most fast and complete dissolution behavior, with a cumulative release of 80.21 ± 0.17% at 5 min and 100.65 ± 0.72% at 120 min. The reason was that the excessive phospholipids in the BVP-PLC with lower drug-lipid ratio (1:3) complexed BVP too tightly which hindered its dissolution. When it came to the BVP-PLC with higher drug-lipid ratio (1:1), phospholipids could not provide strong enough interaction force to complex BVP and maintain the stability of preparation, which resulted the destruction of PLC and the aggregation of BVP in dissolution media and further the compromised dissolution behavior.

### 3.3. Pharmacokinetics Study

#### 3.3.1. Pharmacokinetics Results of Different Preparations: BVP-NS, BVP-LP and BVP-PLC

**Figure 10:**
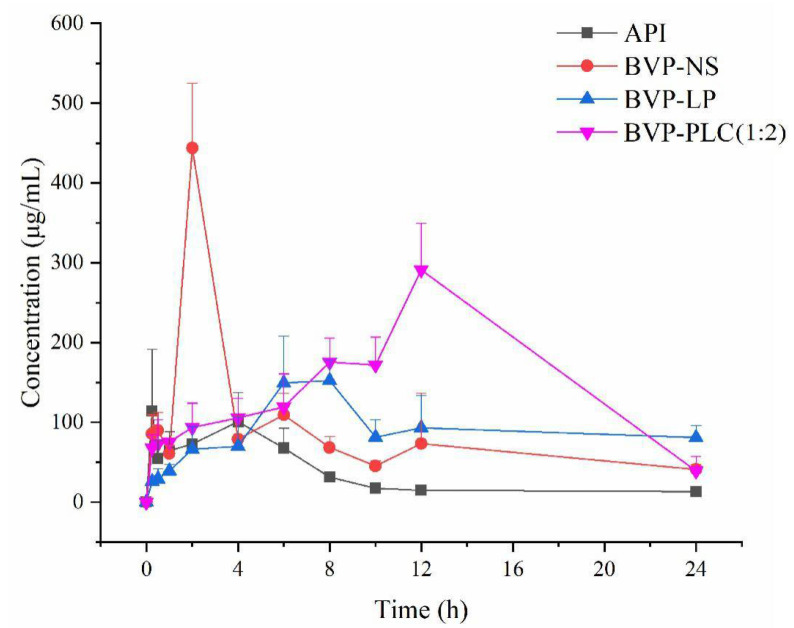
The plasma concentration-time curves of BVP, BVP-NS, BVP-LP and BVP-PLC (1:2) after single administration (i.g.) (*n* = 5).

The pharmacokinetic characteristics of BVP-NS, BVP-LP and BVP-LC (1:2) were investigated in male SD rats after single administration of 20 mg BVP (i.g.). Figure 10 showed the concentration-time curve of different preparations and the specific pharmacokinetic parameters obtained from DAS software were summarized in Table 3. For BVP bulk drug, the plasma drug concentration reached the peak value (101.70 ± 27.45 μg/L) at 3.60 ± 0.89 h (T_max_) with the half life time (t_1/2_) of 3.50 ± 0.53 h and the AUC_(0–t)_ and MRT_(0–t)_ was 803.43 ± 168.33 μg/L·h and 7.30 ± 0.49 h, respectively. The T_max_ of BVP-NS (2.00 ± 0.00 h) was shorter than that of BVP, while the T_max_ of BVP-LP (6.80 ± 1.10 h) and BVP-PLC (1:2) (12.00 ± 0.00 h) were both extended. Compared with BVP, all the three preparations showed different degrees of extending in vivo circulation time, with the MRT_(0–t)_ and t_1/2z_ of 6.70 ± 0.41 h and 4.20 ± 0.76 h for BVP-NS, 10.26 ± 0.74 h and 8.89 ± 1.40 h for BVP-LP, 10.79 ± 0.25 h and 8.95 ± 2.80 h for BVP-PLC (1:2), respectively. As for the maximum concentration, the BVP-NS possessed the highest C_max_ of (444.06 ± 54.10 μg/L), the second for BVP-PLC (291.20 ± 43.08 μg/L) and BVP-LP (166.82 ± 17.72 μg/L) was the lowest one.

As for BVP-NS, it could be seen clearly that the plasma concentration curve rapidly increased to the peak level (444.06 ± 54.10 μg/L) within 2 h and diminished to a relatively low level (153.14 ± 35.69.03 μg/L) at 4 h indicated the rapid absorption and elimination of the BVP-NS in rats. This was highly consistent with the in vitro release behavior, where the most of drug released rapidly in the early stage. In general, the AUC_(0–t)_ of BVP-NS was 1976.22 ± 185.29 μg/L·h which was 2.46 times of BVP. Moreover, mean residence time (MRT_(0–t)_) of BVP-NS had no significant difference (*p* > 0.05) compared with BVP and the overall tendency of drug-time curve were also similar, which indicated the drug in BVP-NS was mainly existed in the original aggregated crystal form. It was worth noting that the T_max_ of BVP-NS (2.00 ± 0.00) was shorter than BVP bulk drug (3.60 ± 0.89) and the C_max_ was much higher, which might attribute to its smaller particle size and greater specific surface area and further the increased contact area with GIT and finally resulted faster absorption and higher oral bioavailability. In addition, a relatively small part of small insoluble particles of BVP-NS could be taken up via microfold (M) cells endocytosis [48], which might further enhance the oral bioavailability.

For the BVP-LP, the plasma drug concentration increased slowly within the first four hours, which still lower than that of BVP. However, it exhibited a relatively fast release at 6 h and reached the maximum concentration at 8 h and then entered in a slowly elimination phase. This characteristic might be related to the particular absorption behavior of liposomes after oral administration [23], whereby the liposomes could partially be destroyed following exposure to gastric acid, digestive enzymes and intestinal surfactants and resulted the release of free BVP. Therefore, the drug-time curve of BVP-LP before 4 h was mainly attributed to the released free BVP. The bilayer structure of liposomes was similar with the cell membrane and therefore liposomes that survived were able to be absorbed via the M cell-to-lymph pathway and epithelium endocytosis which conduced to the plasma drug concentration time curve after 4 h. Most importantly, liposomes absorbed via the M-cell to lymphoid pathway could enter the blood circulation directly without passing through liver metabolism, which could further improve the absorption [23]. These particular absorption mechanisms endowed BVP-LP with extended absorption character and relatively higher oral bioavailability for about 2.38 times of BVP.

**Figure 11:**
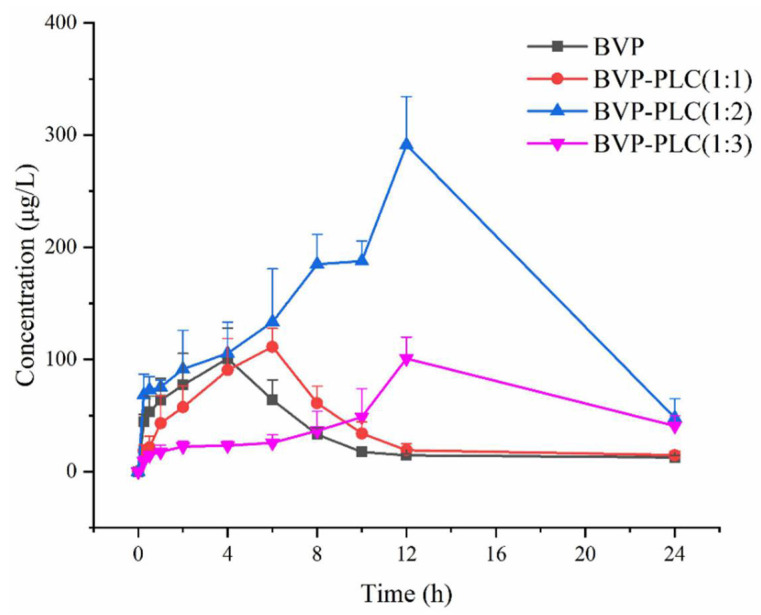
The plasma concentration-time curves of BVP and BVP-PLC with different drug-lipid ratios after single administration (i.g.).

With regard to BVP-PLC (1:2), the plasma drug concentration continued to exhibited an upward tendency and reached the maximum value (291.20 ± 43.08 μg/L) at 12 h. Notably, BVP-PLC (1:2) possessed the supreme AUC _(0–t)_ (3786.72 ± 356.01 μg/L·h) and the relative bioavailability was 4.71 times of BVP, which mainly attributed to its unique absorption mechanism except for the epithelial endocytosis effect. BVP was negatively charged due to its polyphenol structure which ensured the formation of complex with positively charged phospholipid based on electrostatic interaction. In addition, based on previous characterization, there still existed the other form of interaction between BVP and phospholipid (Figure 11). These diverse and relatively strong interactions could maintain the integrity of BVP-PLC (1:2) in GIT and raised possibility for the following absorption process. In specific, BVP-PLC (1:2) could be hydrolyzed to fatty acid and BVP-monoacylglycerol in the presence of phospholipase (especially phospholipase A2) in GIT [49], which could further combine with bile salts and then being absorbed by epithelial cell. The excretion of bile salts is regulated by a hormone called cholecystokinin (CCK), which is sensitive to the concentration of fatty acid. Interestingly, the hydrolysis of BVP-PLC (1:2) could increase the concentration of free fatty acid, which could trigger CCK release and further regulate bile excretion, finally a positive feedback loop kicked in. Once the BVP-monoacylglycerol complex was internalized by epithelial cell in GIT, it would be processed by the enzymes in the smooth endoplasmic reticulum to BVP-diacyl phospholipids. After that, in Golgi apparatus, apolipoprotein B-48 would be integrated to form chylomicrons. Then, BVP loaded chylomicron excreted from epithelial cell through exocytosis and entered in lymphatic circulation, which allowed the BVP-PLC enter in systemic circulation directly and bypass the liver metabolism.

To sum up, BVP-PLC (1:2) was the most promising preparation of the three to address the low bioavailability of BVP. For the BVP-NS, although it could improve the oral bioavailability of the drug and had highest C_max_ of the three, the fast systemic metabolism would hinder its further application. Therefore, the main task of BVP-NS was to extend the release of drug and prolong the circulation time in vivo. Some researchers have performed relevant studies in this field, Peng Ji et al. modified curcumin nanocrystals with hyaluronic acid, which raised the blood elimination half-life from 14.66 ± 6.58 h to 53.06 ± 18.21 h [50]. Although BVP-LP could significantly extend the mean retention time and half-life time, the relative bioavailability was not enhanced greatly compared with BVP-NS. Phospholipid complexes could both significantly increase the oral bioavailability and prolong the half-life time of the drug. Notably, in vitro release study showed that the drug-lipid ratio had remarkable influence on its dissolution behavior. Therefore, the pharmacokinetics of the BVP-PLC with different drug-lipid ratios were studied.

#### 3.3.2. Pharmacokinetic Results of BVP-PLC with Different Drug-Lipid Ratios

According to the in vitro release results, the drug-lipid ratios had significant influence on dissolution behavior, therefore the corresponding pharmacokinetic study was also carried out and the concentration-time curve and pharmacokinetic parameters were shown in Figure 11 and Table 4, respectively. For the BVP-PLC (1:1), almost all pharmacokinetic parameters (AUC_(0–t)_, AUC_(0–∞)_, MRT_(0–∞)_, t_1/2_, C_max_) showed no significant difference compared with BVP except for the MRT_(0–t)_ (8.03 ± 0.31 h) and T_max_ (5.60 ± 0.89 h) with a little prolonged and the relative bioavailability was 118.06% which was also not greatly improved. The BVP-PLC with 1:2 drug-lipid ratio possessed the highest relative bioavailability of BVP (471.32%) and the maximum T_max_ (12.00 ± 0.00 h) and C_max_ (291.20 ± 43.08 μg/L), while with the moderate MRT_(0–t)_ (10.79 ± 0.25 h) and t_1/2_ (8.95 ± 2.80 h). With regard to the BVP-PLC with drug-lipid ratio of 1:3, its AUC_(0–t)_ was significantly enhanced for about 1.57 times of BVP and possessed the same T_max_ with BVP-PLC (1:2) and the longest MRT_(0–t)_ (12.99 ± 0.71 h) and t_1/2_ (17.67 ± 3.63 h), while the smallest C_max_ (100.95 ± 19.00 μg/L) among the three. Therefore, it could be concluded that the drug-lipid ratio indeed had significantly influence on the in vivo process. In specific, the lower drug-lipid ratio was conducive to the longer circulation time in vivo, while the relative bioavailability possessed an entirely different tendency. As could be seen in Table 4, BVP-PLC (1:2) had the highest relative bioavailability and second for BVP-PLC (1:3), the last for BVP-PLC (1:1).

This unusual tendency might attribute to the distinct interaction type and strength between drug and phospholipids in the BVP-PLC with different drug-lipid ratios, which further resulted in disparate preparation stability, drug absorption in GIT and release behavior in vivo. Through molecular simulation, it was found that there are mainly two kinds of forces (hydrogen bond and electrostatic interaction). For BVP-PLC (1:1), the molar ratio of drug to phospholipid was about 2:1 or even higher. So, the relatively less phospholipid molecule could not provide strong enough interaction forces to complex BVP and maintain the stability of preparation, which resulted its structure was very easy to be destroyed in GIT and the aggregation of drug, therefore its pharmacokinetic behavior was similar to BVP bulk drug and the AUC_(0–t)_ had no significant difference. When the drug-lipid ratio raised to 1:2, the molar ratio of phospholipid and drug was about 1:1. Based on the previous results, this ratio might be the most satisfactory prescription, which could provide moderate interaction to exactly balance the drug loading and drug release. On the one hand, the BVP-PLC at this drug-lipid ratio could maintain the integrity of the complex structure in GIT, which ensured the relatively higher absorption. On the other hand, this suitable interaction could also make the drug release more complete and thereby the oral bioavailability (471.32%) was improved greatly. As for drug-lipid ratio of 1:3 or even lower, the phospholipid in preparation was so overwhelmed that the encapsulation forces were too tightly, which hindered the release of BVP from the preparation and leading to a relatively lower relative bioavailability (157.00%). Therefore, it could be concluded that the drug-lipid ratio played a very important role in oral bioavailability of BVP-PLC, the more suitable drug-lipid ratio, the higher oral bioavailability.

## 4. Conclusions

The absorption process, in vivo stability and release profile are all very important factor to the oral bioavailability of BVP. In this study, three preparations, BVP-NS, BVP-LP and BVP-PLC were investigated. The pharmaceutical study indicated that the particle size of the three preparations was in the range of 200–300 nm and the zeta potential was all negative. The encapsulation efficiency of BVP-LP was 76.41 ± 5.61% and the complexation efficiency of BVP-PLC (1:2) was 72.90 ± 3.21%. PLM, PXRD and DSC results suggested that BVP was presented as crystal in nanosuspension, while as non-crystal form in liposome and phospholipid complex. FI-IR experiment demonstrated that the –OH and –COO^−^ group in BVP could interact with phospholipid molecule during phospholipid complex formation, rather than nanosuspension and liposome. Molecular simulation further confirmed that hydrogen bond and electrostatic interaction were the two main kinds interactions. In vitro release results manifested that BVP-PLC (1:2) showed the highest cumulative release amount of 100.65 ± 0.72%, followed by BVP-NS (63.77 ± 3.58%) and BVP-LP (51.50 ± 1.32%). In vivo pharmacokinetic study verified that BVP-NS, BVP-LP and BVP-PLC all shown significantly higher oral bioavailability compared with BVP, with the relative bioavailability of 245.97%, 237.51% and 471.32%, respectively. In addition, BVP-LP and BVP-PLC exhibited a distinctly extended-release profile with the MRT_(0–t)_ of 10.26 ± 0.74 and 10.79 ± 0.25, compared with BVP (7.30 ± 0.49). Furthermore, the drug-lipid ratio had significant influence on the pharmacokinetic parameters of BVP-PLC, in which the preparation with drug-lipid ratio of 1:2 had the highest relative bioavailability (471.32%). As the higher drug-lipid ratio (1:1) could not provide strong enough force to maintain the stability of the preparation and the lower drug-lipid ratio (1:3) impacted the dissociation of BVP from the preparation. Therefore, phospholipid complexes with appropriate drug-lipid ratio provide an effective strategy for improving the oral bioavailability of drugs which have low solubility both in water and lipid.

## Figures and Tables

**Table 1:** UPLC-MS/MS gradient elution method for BVP analysis.

Time (min)	Flow Rate (mL/min)	%A	%B
0.0	0.2	60	40
0.5	0.2	60	40
1.2	0.2	25	75
1.8	0.2	5	95
2.8	0.2	5	95
3.0	0.2	60	40
3.5	0.2	60	40

**Table 2:** Mean particle size, PDI, CE%, EE% and zeta potential/mV of three preparations.

Formulation	Drug-Lipid Radio	Mean Particle Size/nm	PDI	EE%/CE%	Zeta Potential/mV
BVP-NS	-	277.4 ± 5.6	0.234 ± 0.070	-	−15.2 ± 0.6
BVP-LP	-	265.3 ± 13.0	0.221 ± 0.058	76.41 ± 5.61	−13.1 ± 1.3
BVP-PLC	1:1	272.2 ± 20.1	0.29 ± 0.068	61.31 ± 2.32	−20.5 ± 0.8
BVP-PLC	1:2	298.6 ± 36.6	0.278 ± 0.051	72.90 ± 3.21	−22.2 ± 1.0
BVP-PLC	1:3	338.4 ± 26.7	0.266 ± 0.047	79.36 ± 3.76	−21.0 ± 0.9

**Table 3:** Pharmacokinetics parameters of BVP, nanosuspensions, liposomes and complexes (equivalent to BVP 20 mg/kg).

Parameter	Unit	BVP	BVP-NS	BVP-LP	BVP-PLC (1:2)
AUC_(0–t)_	μg/L·h	803.43 ± 168.33	1976.22 ± 185.29 **	1908.26 ± 316.62 **	3786.72 ± 356.01 **
AUC_(0–∞)_	μg/L·h	810.65 ± 171.45	2007.77 ± 194.50 **	2422.41 ± 491.91 **	4460.31 ± 597.51 **
MRT_(0–t)_	h	7.30 ± 0.49	6.70 ± 0.41	10.26 ± 0.74 **	10.79 ± 0.25 **
MRT_(0–∞)_	h	9.57 ± 0.59	9.41 ± 1.07	15.83 ± 2.36 **	14.75 ± 2.75 *
t_1/2z_	h	3.50 ± 0.53	4.20 ± 0.76	8.89 ± 1.40 **	8.95 ± 2.80 **
T_max_	h	3.60 ± 0.89	2.00 ± 0.00 *	6.8 ± 1.10 **	12 ± 0 **
C_max_	μg/L	101.70 ± 27.45	444.06 ± 54.10 **	166.82 ± 17.72 **	291.20 ± 43.08 **
Relative Bioavailability		-	245.97%	237.51%	471.32%

All the data were presented in the form of mean ± SD, *n* = 5. * indicates a statistically significant difference between BVP and BVP-NS, BVP-LP or BVP-PLC (1:2) (*p* < 0.05); ** indicates *p* < 0.01 vs. BVP.

**Table 4:** Pharmacokinetics parameters of BVP and BVP-PLC with different drug-lipid ratios (equivalent to BVP 20 mg/kg).

Parameter	Unit	BVP	1: 1	1: 2	1: 3
AUC_(0–t)_	μg/L·h	803.43 ± 168.33	948.50 ± 70.46	3786.72 ± 356.01 **	1261.41 ± 124.99 **
AUC_(0–∞)_	μg/L·h	810.65 ± 171.45	1002.07 ± 90.48	4460.31 ± 597.51 **	2327.07 ± 321.38 **
MRT_(0–t)_	h	7.30 ± 0.49	8.03 ± 0.31 *	10.79 ± 0.25 **	12.99 ± 0.71 **
MRT_(0–∞)_	h	9.57 ± 0.59	10.47 ± 0.83	14.75 ± 2.75 *	29.72 ± 6.56 **
t_1/2z_	h	3.50 ± 0.53	4.69 ± 2.82	8.95 ± 2.80 **	17.67 ± 3.63 **
T_max_	h	3.60 ± 0.89	5.60 ± 0.89 **	12.00 ± 0.00 **	12.00 ± 0.00 **
C_max_	μg/L	101.70 ± 27.45	117.22 ± 17.73	291.20 ± 43.08 **	100.95 ± 19.00
Relative Bioavailability		-	118.06%	471.32%	157.00%

All the data were presented in the form of mean ± SD, *n* = 5. * indicates a statistically significant difference between BVP and BVP-PLC (*p* < 0.05); ** indicates *p* < 0.01 vs. BVP.

## Data Availability

The data presented in this study are available on request from the corresponding author. The data are not publicly available due to privacy.
